# Preparation and Investigation of Foaming Amphiphilic Fluorinated Nanoparticles for Enhanced Oil Recovery

**DOI:** 10.3390/ma10121403

**Published:** 2017-12-08

**Authors:** Keliang Wang, Gang Wang, Chunjing Lu, Cuiying Pei, Ying Wang

**Affiliations:** 1 Key Laboratory for EOR Technology (Ministry of Education), Northeast Petroleum University, Xuefu Road 99, Daqing 163318, China; s1203268@stu.nepu.edu.cn (K.W.); chunjinglu@outlook.com (C.L.); 2 Center for High Pressure Science and Technology Advanced Research, Cailun Road 1690, Shanghai 201203, China; cuiyingp@outlook.com; 3 State Key Laboratory of Inorganic Synthesis & Preparative Chemistry, Jilin University, Qianjin Road 2699, Changchun 130012, China; ywang0707@outlook.com

**Keywords:** amphiphilic nanoparticles, anisotropy, colloid surfactants, enhanced oil recovery

## Abstract

Amphiphilic nanoparticles have attracted increasing interest as Pickering emulsifiers owing to the combined advantages of both traditional surfactants and homogeneous particles. Here, foaming amphiphilic fluorinated nanoparticles were prepared for enhanced oil recovery by the toposelective surface modification method. The structure and properties of amphiphilic nanoparticles were characterized using Fourier transform infrared spectroscopy, scanning electron microscopy, a laser diffraction method, fluorescence microscopy, a pendant drop tensiometer, and foamscan. It was found that the amphiphilic fluorinated nanoparticles exhibited significant interfacial activity at the air–water interface and generated stabilized aqueous foams against coalescence and drainage even in the absence of surfactants. When the particle concentration reached 0.6 wt %, the adsorption of the amphiphilic nanoparticles at the interface was saturated and the equilibrium surface tension dropped to around 32.7 mN/m. When the particle concentration reached 0.4 wt %, the Gibbs stability criterion was fulfilled. The amphiphilic nanoparticles foam system has a better plugging capacity and enhanced oil recovery capacity. The results obtained provide fundamental insights into the understanding of the self-assembly behavior and foam properties of amphiphilic fluorinated nanoparticles and further demonstrate the future potential of the amphiphilic nanoparticles used as colloid surfactants for enhanced oil recovery applications.

## 1. Introduction

Aqueous foams are of great practical interest because of their extensive applications, which range from cosmetics and food, to fire extinguishing, mineral flotation processing, and oil recovery [1,2,3,4,5]. The application of foam in oil recovery faces some technical and economic challenges, for instance, it is a source of gas, with high cost, and the surfactant absorbs on the rock; the biggest problem is the stability of foam fluid [6,7,8]. Foam instability, which is derived from liquid drainage, disproportionation and coalescence in films, is a critical issue in all these applications. Although destabilization mechanisms can be partially hindered by using low molecular weight surfactants that are easily detached from the interface, these systems suffer from low stability [9]. In addition to surfactants, solid particles are a promising alternative for enhancing foam stability [10,11,12,13,14].

The attachment of particles at the gas-liquid interface forms a close-packed structure and creates a steric barrier to effectively inhibit the destabilization of foams caused by drainage and the diffusion of gas [15]. Contrary to low molecular weight surfactants, colloidal particles can be effectively and irreversibly adsorbed at the interface given that their desorption energy levels are in the thousands, thereby making them extremely effective as foam stabilizers [16,17,18,19]. In general, particle-stabilized foams inhibit bubble growth and drainage over a period of days or weeks as compared to the stability period of surfactant-stabilized bubbles, which is generally less than a few hours [10,13].

At present, the study of foam stabilized by nanoparticles for enhancing oil recovery mainly includes homogeneous modified particles and mixtures of particles and surfactants. The homogeneous particles with uniform surface wettability have been employed for Pickering stabilization. The adsorption of the homogeneous particles at the gas–liquid interface mainly depends on their size and wettability, which determines the contact angle θ of the particles at the interface [20,21,22]. Utilization of the homogeneous particles for the preparation of Pickering emulsions requires large amounts of energy, which are generally derived from strong shear or ultrasonication, to force the particles at the interface to pass the energy barrier [23]. In the mixtures of particles and surfactants, a similar synergistic effect exists among different surfactants [24,25,26]. Compared to a sole particle or surfactant, both the foaming ability and foam stability of the mixtures improve substantially. However, the surface activity of the mixtures significantly decreases at high temperature, which makes the mixtures lose their synergistic effect. Therefore, the question concerns the ability of amphipathic particles to overcome the energy barrier and enhance their kinetics, thereby allowing their spontaneous adsorption onto the interface without the necessity of excess shear. Amphipathic particles can combine their anisotropic wettability and high interfacial activity with the Pickering character to irreversibly adsorb to the interface. Very recently, further progress has been made in the application of amphipathic particles as solid surfactants to effectively stabilize emulsions and achieve longer-term stability as compared to those stabilized by low molecular weight surfactants and homogeneous particles [27,28,29,30,31,32,33,34]. Nevertheless, the amphiphilic fluorinated silica nanoparticles are rarely synthesized for the stabilization of the air–water surface.

In this article, we developed the tailored synthesis of fluorinated Janus particles (named after the roman god Janus) which provide asymmetry, and can thus impart drastically different chemical or physical properties and directionality within a single particle. The properties of hydrocarbons and, therefore, surfactants, were significantly altered following the substitution of fluorine atoms with hydrogen atoms [35]. The substitution decreased their surface activity in aqueous solutions, thereby lowering the critical micelle concentration and surface tension relative to that of other hydrocarbon analogues [36]. In addition, the fluorinated chains were more thermally stable than their corresponding alkyl chains due to the high energy of their C–F bonds (466 kJ/mol), thereby maintaining the intactness of the fluorinated compounds in hydrocarbon analog-degrading environments [37]. Additionally, the fluorinated compounds improved the oil tolerance of the foam [38]. We investigated the effect of the fluorinated amphipathic particle concentrations on the adsorption behavior at the air-water interface as well as the stability of the foam, to reveal the underlying foam stabilization mechanisms. Additionally, the plugging capacity and oil displacing capacity of four foam systems were systematically investigated, consisting of traditional surfactant, homogeneous modified particles, mixtures of particles and surfactants and amphiphilic particles.

## 2. Materials and Methods

### 2.1. Materials

Fumed silica particles (200 nm in diameter) were purchased from the Sinopharm Chemical Reagent Company (Shanghai, China). Paraffin (melting temperature range: 55–57 °C) was purchased from the Shanghai Huayong Paraffin Company (Shanghai, China). (3-aminopropyl)triethoxysilane (APTES) (99%), pentadecafluorooctanoic acid (PFOA), didodecyldimethylammonium bromide (DDAB), fluorescein isothiocyanate (FITC), phosphate buffer solution (PBS), and dimethyl sulfoxide (DMSO) were purchased from Sigma Aldrich (St. Louis, MO, USA). Fumed silica particles with various degrees of hydrophobicity were received in powder form and were gifts from Wacker Chemie (Munich, Germany): T30 (180 nm, 100% Si–OH coverage), H30 (195 nm, 50% methylsilyl capped, 50% Si–OH). All products were used as received.

### 2.2. Preparation of the Amphiphilic Fluorinated Nanoparticles

**Figure 1:**
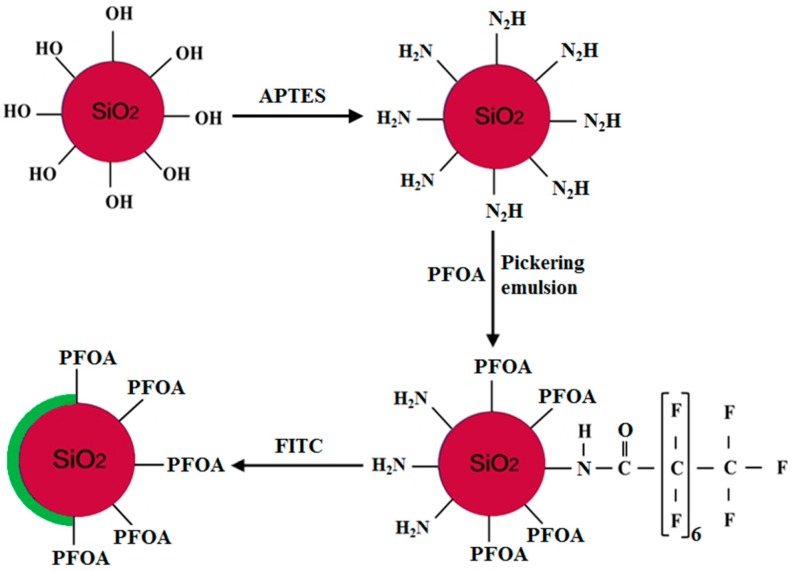
Synthesis scheme of the fluorescent Janus particles by the Pickering emulsion approaches.

Scheme of the synthesis of the amphiphilic fluorinated nanoparticles is illustrated in Figure 1. In a typical silanization procedure, 5 wt % APTES was hydrolyzed in a 2 wt % ethanol solution and magnetically stirred for 10 min. Subsequently, 0.2 wt % silica particles were dispersed in the above mixture and stirred for 24 h in a nitrogen atmosphere. The solution was then centrifuged and rinsed by anhydrous ethanol several times to remove the unreacted APTES. Moreover, the modified silica particles were dried in a vacuum oven at 70 °C for 12 h, and used for the next step of the experiment and for further characterization.

Firstly, 0.4 g silica particles modified with APTES were dispersed in 400 mL deionized water and magnetically stirred at 3000 rpm for 15 min at 70 °C. Next, 45 g wax was added to the above mixture and stirred at 3500 rpm for 45 min. The produced emulsions were immediately cooled in an ice bath without stirring to solidify the wax. The solid wax colloidosomes were washed multiple times with deionized water to remove the unattached particles. After the colloidosomes were completely dried under a vacuum at 40 °C, the solid paraffin droplets were dispersed in a PFOA–water solution for 72 h at room temperature under stirring. Thereafter, the colloidosomes were filtrated and washed several times with deionized water to remove the unreacted PFOA and free particles. The colloidosomes were then dissolved in chloroform to release the modified silica particles. Finally, the amphiphilic fluorinated particles were recovered by centrifugation and dried under a vacuum for 48 h.

### 2.3. Preparation of the Fluorescent Amphiphilic Fluorinated Nanoparticles

Scheme of the synthesis of the fluorescent amphiphilic fluorinated nanoparticles is illustrated in Figure 1. A solution of FITC (10 mg) in an acetone/water mixture (1/9, *v*/*v*) was added to a dispersion of Janus particles (20 mg) in PBS (20 mL, pH 7.5). The mixture was stirred for 12 h in a dark environment at room temperature. Following FITC tagging, the Janus particles were centrifuged and washed three times with PBS to remove the unreacted fluorescent agent, and the FITC-tagged Janus particles were suspended in DMSO for fluorescence microscopy.

### 2.4. Characterization of the Modified Nanoparticles

The surface morphologies of the colloidosomes were observed by scanning electron microscopy (SEM, JEOL Co., Tokyo, Japan). Bare and modified silica particles in pellet form were generated following the addition of spectroscopic grade KBr. The Fourier transform infrared spectroscopy (FTIR) spectra were recorded using a 1615 FTIR spectrometer (PerkinElmer, Waltham, MA, USA). The particle size distribution of the Janus particles was measured by a laser diffraction method (Mastersizer 2000, Malvern Instruments Ltd., Malvern, UK). The fluorescent amphiphilic nanoparticles and foams were optically analyzed using a FV3000 microscope (Olympus, Tokyo, Japan) with an electron CCD camera.

### 2.5. Characterization of the Interfacial Properties

The surface tension ϒ and the dilational elastic modulus E of the amphiphilic nanoparticles were measured using a pendant drop tensiometer (Longessaigne, Teclis, France). The syringe was filled with a dispersion of amphiphilic nanoparticles for a range of concentrations. In this method, the software-controlled syringe allowed volume drop changes to generate a sinusoidal profile. The recorded shape of the pendant drop was analyzed to obtain the surface tension.

### 2.6. Characterization of the Foaming Properties

The foam stability and foam drainage were determined using foamscan (Longessaigne, Teclis, France). An amount of 25 mL of the foaming suspension at a range of different Janus particle concentrations was foamed by sparking N_2_ through a porous disk at a constant gas flow rate of 100 mL/min. Foam generation halted when a foam volume of 150 mL was obtained. The liquid fraction in the foam column was measured using a couple of electrodes.

### 2.7. Plugging Capacity

**Figure 2:**
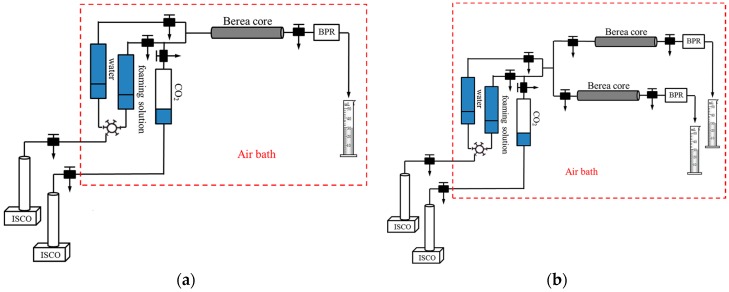
Schematic diagram of (**a**) the single Berea core flooding experiments and (**b**) the parallel Berea core flooding experiments.

Single Berea sandstone core experiments were used to determine the plugging capacity of four foam systems, respectively. The composition of the four foam systems was shown in Table 1. The experimental apparatus was shown in Figure 2a. The length of Berea sandstone core was 30 cm and the inside diameter was 4.5 cm. The steps were conducted as follows: (1) Vacuumize the core for 4 h; (2) Saturate the core with brine, then calculate permeability and pore volume. The pressure in this step was recorded P_1_; (3) Inject 1.0 pore volume (PV) of foaming solution into the core. The pressure in this step was recorded P_2_; (4) Flood water until the injection pressure becomes stable. The pressure was recorded P_3_ in this process. In these single sand-pack experiments, the resistance factor was calculated as P_2_/P_1_ or P_3_/P_1_ to characterize the plugging capacity of these four foam systems.

### 2.8. Enhanced Oil Recovery

Parallel Berea sandstone core experiments were conducted to determine the enhanced oil recovery of these four foam systems. The experimental apparatus is shown in Figure 2b. The length of the Berea sandstone core was 30 cm and the inside diameter was 4.5 cm. The steps are as follows: (1) Vacuumize the core for 4 h; (2) Saturate the core with brine, then calculate the pore volume; (3) Saturate oil and age for 24 h at 80 °C; (4) Flood water until the effluent water cut is over 98%; (5) Inject 1.0 PV of foaming solution into the parallel Berea sandstone core; (6) Flood water until the effluent water cut is over 98% again. The oil production and water production data were recorded to analyze the enhanced oil recovery of these four foam systems.

## 3. Results

### 3.1. Characterization of the Amphiphilic Fluorinated Nanoparticles

**Figure 3:**
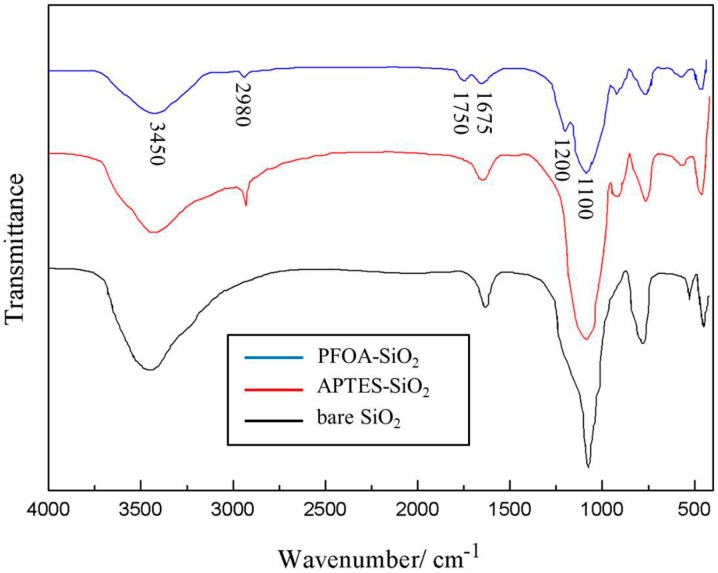
Fourier transform infrared (FTIR) spectra of silica particles.

The FTIR spectra of the bare and modified silica particles are illustrated in Figure 3. In the case of the bare silica particles, the sharp band at 3450 cm^−1^ corresponded to the presence of silanol groups (Si–OH) on the silica surface. The absorption bands at 1675 cm^−1^ and 1100 cm^−1^ are related to the bending vibration of H_2_O and the isolated terminal silanol (Si–OH) groups, respectively. The spectra of the silica particles modified with APTES exhibited weakened silanol groups (Si–OH) and H_2_O absorption bands, which may have been a result of the APTES modification. Moreover, the spectra of the modified particles manifest a band at 2980 cm^−1^, which is attributed to the asymmetric stretching of the C–H bond due to the presence of aminopropyl groups. As for the amphiphilic fluorinated nanoparticles, the absorption bands at 1200 and 1750 cm^−1^, which are assigned to the stretching vibration of –C–F– and C=O, indicated the presence of the PFOA layer in the fluorinated particles.

**Figure 4:**
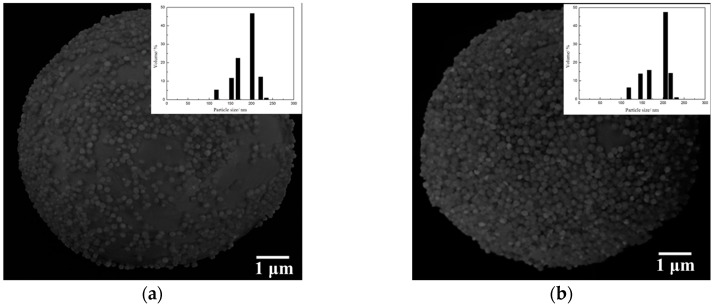
SEM images of the wax colloidosomes stabilized by (**a**) the bare silica and (**b**) the APTES-silica. The insets show the size distribution of the silica particles.

In the synthesis process of Janus particles by the Pickering emulsions method, the silica particles assembled on the oil–water interface were immobilized on the surface of the wax colloidosomes during wax solidification. The colloidosomes structure was analyzed by SEM, as presented in Figure 4. The silica particles modified with APTES packed more closely and orderly on the surface of the wax colloidosomes compared to the bare ones, which markedly increased the efficiency of the modification process. In addition, there was no significant difference in particle size distribution between the bare and modified silica particles.

**Figure 5:**
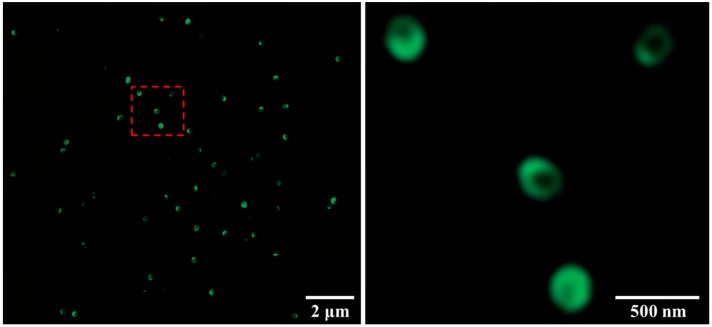
Fluorescence images of the fluorinated silica Janus particles with fluorescein isothiocyanate (FITC).

As shown in Figure 5, one side of the particles labeled with FTIC was always more green-fluorescent than the other one decorated with fluorinated groups using fluoroalkylsilane. The slight fluorescence emerged on the fluorinated side of the particles because of multiple reflections and scatterings of the illuminating light emitted by the fluorophore on the edges of transparent silica particles.

### 3.2. Interfacial Properties of the Amphiphilic Fluorinated Nanoparticles

We studied the behavior of the air–water interface in the presence of the amphiphilic nanoparticle dispersions. In the first part, we determined the time evolution of the air–water surface tension ϒ (dynamic surface tension) at different amphiphilic nanoparticle concentrations. In the second part, we plotted the interfacial dilatational elasticity (E) against the dynamic surface tension ϒ(t), which is associated with foam stability.

**Figure 6:**
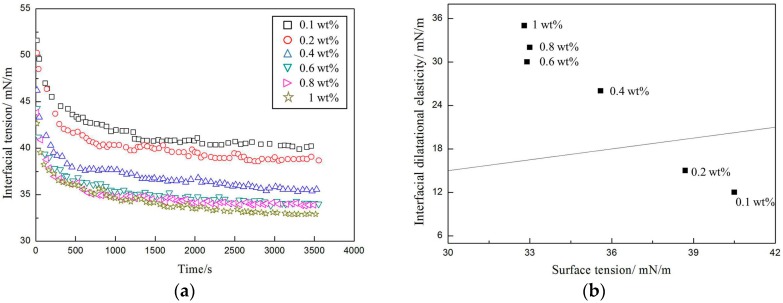
The interfacial properties of the amphiphilic fluorinated nanoparticles at various concentrations: (**a**) Dynamic surface tension; (**b**) Variation of E with the surface tension ϒ. The solid line shows E = ϒ/2 (Gibbs stability criterion).

According to Figure 6a, the surface tension dropped rapidly in the early stages and then reached equilibrium at different observed concentrations. At higher amphiphilic nanoparticle concentrations, the surface interfacial tension rate decreased and reached a lower equilibrium level. Additionally, as the amphiphilic nanoparticle concentration increased, the equilibrium surface tension kept decreasing until it reached the critical concentration, at which point the adsorption of the amphiphilic nanoparticles at the air–water interface was saturated. At the critical concentration (0.6 wt %), the equilibrium surface tension dropped to around 32.7 mN/m. A dilational elastic modulus of E > ϒ/2, also called the Gibbs stability criterion, is a foam stability evaluation criterion [18]. According to Figure 6b, the Gibbs stability criterion was fulfilled for dispersions with amphiphilic nanoparticle concentrations greater than 0.2 wt %.

### 3.3. Foaming Properties of the Amphiphilic Fluorinated Nanoparticles

**Figure 7:**
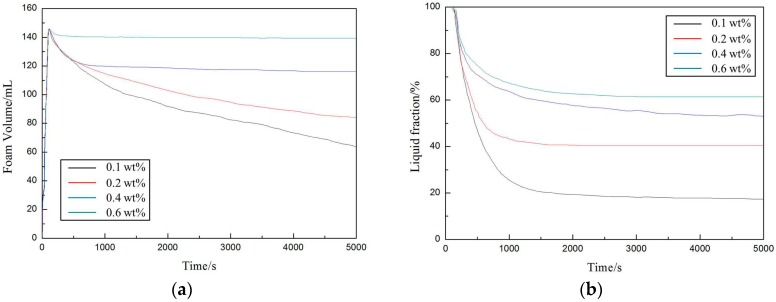
(**a**) Foam stability and (**b**) Liquid foam fraction of the amphiphilic fluorinated nanoparticles at various concentrations.

The time-dependence of the foam and liquid volumes at different foam concentrations was also investigated, as shown in Figure 7.

According to Figure 7a, higher amphiphilic nanoparticle concentrations resulted in slower foam collapses and higher foam stability values. Amphiphilic nanoparticle concentrations above 0.2 wt %, which fulfilled the Gibbs stability criterion, exhibited almost constant foam volumes during the observation period. According to Figure 7b, the liquid fraction decreased until it reached a constant level during the drainage process. The finial liquid volume, which increased as the amphiphilic nanoparticle concentrations increased, significantly affected the stabilization of the foams.

### 3.4. Plugging Capacity

**Figure 8:**
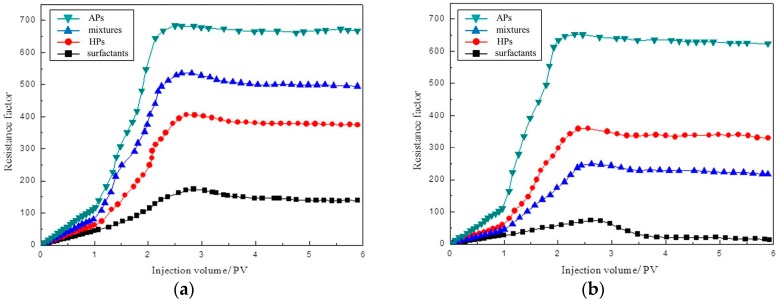
Plugging capacity of four foam systems at (**a**) 40 °C and (**b**) 80 °C.

Figure 8 shows the plugging capacity of four foam systems under the reservoir condition of 40 °C, and 80 °C. The core permeability is 10 μm^2^, and the injection rate is set as 0.6 mL/min. The results of Figure 8 express that the resistance factors of the amphiphilic particles foam system is higher than other foaming systems under two temperature conditions; it also shows superior plugging capacity. The amphiphilic particles foam system can stabilize the foam better under two temperatures. Under the high temperature of 80 °C, the plugging capacity of foam stabilized by amphiphilic particles reveals more outstanding advantages. The resistance factors of surfactants and the mixture of nanoparticles and surfactants decreased significantly at 80 °C as compared to those at 40 °C.

### 3.5. Enhanced Oil Recovery

The enhanced oil recovery of four foam systems is shown in Table 2. The experimental data of amphiphilic particles foam flooding show that the enhanced oil recoveries of the low permeability physical model and the high permeability physical model are 49.43% and 16.31% respectively, and the ultimate oil recovery is 67.76% which improves by 32.87% after injection of the foam systems; this represents the highest increased range of enhanced oil recovery of the four foam systems.

## 4. Discussion

### 4.1. Preparation of the Amphiphilic Fluorinated Nanoparticles

In the synthesis process of the Janus particles by the Pickering emulsions method, although the original method described by Granick et al. [39] also facilitates the migration of the silica particles towards the oil–water interface by increasing the surfactant concentration, the excess of surfactants reduced the amount of available functional groups (Si–OH) that acted as anchors to the silane coupling agents, thereby significantly decreasing the efficiency of the modification stage [40]. The wetting response is a common result of the transformation of the functional groups on the particle surface and variations in the surface roughness of modified silica particles. The terminal silanol groups on the silica particle surfaces were substituted by more hydrophobic amino groups which enhanced the hydrophobicity of the silica particles. Additionally, silica particles modified with APTES exhibited lower surface-free energy values (ϒ_APTES_ = 49 mJ/m^2^) as compared to those of the bare silica particles (ϒ_SiO2_ = 72 mJ/m^2^). Therefore, the modified silica particles possessed a higher affinity to the hydrophobic wax (ϒ_wax_ = 32 mJ/m^2^) [41]. According to the Wenzel model for a hydrophilic surface with a Young’s contact angle of <90°, the hydrophilicity is expected to increase following an increase in surface roughness as the liquid penetrates the roughness features [42,43]. Although a silane layer may build a hierarchical nano/micro structure (surface roughness), the difference in the contact angle is mainly a result of the transformation of the functional groups on the particle surface. Fluorescence microscopy (Figure 5) verified the asymmetric surface modification of the obtained Janus particles. This not only confirmed the Janus character of the particles but also showed that silica particle surfaces originally shielded by wax were still active after further chemical modifications.

### 4.2. The Interfacial Properties and Foaming Properties of the Amphiphilic Fluorinated Nanoparticles

The concentration of amphiphilic nanoparticles, which fulfilled the Gibbs stability criterion, was significantly lower than that of the homogeneous particles reported by Stocco et al. [18]. The adsorption behavior of the amphiphilic nanoparticles oriented at the air–water interface was drastically different from that of the homogeneous particles. The amphiphilic nanoparticles exhibited a negative free energy to form a particle–coated interface, thereby allowing the amphiphilic nanoparticles to overcome the energy barrier formed by steric and/or electrostatic interactions. This result demonstrates the applicability of the surface chemical composition asymmetry for spontaneous assembly at the air–water interface. The foams stabilized by the amphiphilic nanoparticles exhibited almost constant foam volumes during the observation period, which was much longer than the foams prepared with low molecular weight surfactants that generally exhibited stability for only a few hours. In addition, the liquid fraction of the foams stabilized by the amphiphilic nanoparticles will reach a constant level during the drainage process. On the contrary, low molecular weight surfactants at all concentrations did not reach static equilibrium [44].

### 4.3. The Plugging Capacity and Enhanced Oil Recovery

Generally, the traditional aqueous foam is loose and non-uniform, which makes it easily defoamed when transporting in porous media. Meanwhile, the aging in porous media also intensifies the instability of foam, and reduces the plugging capacity in the water flooding stage. Since the surface activity of surfactant significantly decreases under the high temperature of 80 °C, the stability of foam decreases sharply. The resistance factors of the homogeneous modified particles foam system and amphiphilic particles foam system have no significant change under two temperatures, and the adsorption-free energy of particle absorption on the interface is higher compared to that of surfactant adsorption, which is more difficult to detach from the interface. Homogeneous modified particles have emerged as an important material to produce stable CO_2_ foam for enhanced oil recovery. Worthen et al. [45] have shown the use of nanoparticles with the proper hydrophilic/CO_2_-philic balance (HCB) as foam stabilizers offers the possibility of generating highly stable CO_2_ foams without the use of surfactants or polymers. Yu et al. [46] reported the effect of the wettability of silica nanoparticles on CO_2_ foams generation and foam flow behavior in porous media. As the nanoparticle surface property changed from hydrophilic to somewhat hydrophobic, more CO_2_ foam was observed to generate. However, the dispersion of the homogeneous modified nanoparticles with a certain degree of hydrophobicity is poor in water, which is the main handicap to be widespread use in stabilizing foam for oil recovery. Compared to a simple particle or surfactant, a foam system containing a mixture of particles and surfactants shows a synergistic effect similar to that of different types of surfactants, and the foaming ability and foam stability of the mixtures are improved substantially. Singh and Mohanty [47] investigated the synergistic stabilization of foam with a mixture of nanoparticles and anionic surfactant in the Berea sandstone cores. They demonstrated that, by adding a small amount of nanoparticles to the surfactant solutions, the foam stability dramatically increased, which was evident from the high resistance factor achieved in their foam-flow experiments. Worthen et al. [48] reported the generation of viscous and stable CO_2_ foams with fine texture by use of bare silica nanoparticles and zwitter–ionic surfactant in beadpacks, when neither of these species could stabilize foam independently. However, the surface activity of surfactant is inactive at high temperature, and the hydrophilic particles in the system can hardly stabilize the foam separately. Therefore, only bridging between particles can be adopted to seal the pore throats, reduce core permeability and significantly decrease the resistance factor (Figure 8b). The amphiphilic particles foam system can stabilize the foam better under two temperatures; this is because the asymmetry of wettability can generate its owns surface activity and actively adsorbs to the gas–water surface. Moreover, the inherent attribute of inorganic particles can make them have high desorption energy at the interface, which shall be deemed as irreversible adsorption.

The enhanced oil recovery of four foam systems indicates that the foam can effectively improve the flowing behavior of fluids in a heterogeneous reservoir, and the foam system can exert its unique effect of heterogeneity to preferably improve oilfields in a heterogeneous reservoir compared to a homogeneous reservoir. First of all, the foam enters into the pore path with high permeability, and then it will mainly enter into low permeability areas through direct plugging and bridging as well as the Jamin effect. Nevertheless, the oil recovery is very different. The recovery capacity enhanced by the amphiphilic particles foam system is much better than the recovery capacity of other foam systems. These differences are because amphiphilic particles not only generate their own surface activity but also stably absorb inorganic particles at the interface. The amphiphilic particles foam system is much easier to blister in the core, and the generated foam is much more stable. Hence, the amphiphilic particles foam system can effectively improve profile control and oil recovery.

## 5. Conclusions

The present study aimed to synthesize Janus particles using partially grafting fluorinated chains as foam stabilizers. These particles exhibited significant interfacial activity at the air–water interface and generated stabilized aqueous foams against coalescence and drainage even in the absence of surfactants. As the interface attained saturation adsorption, the equilibrium surface tension dropped to around 32.7 mN/m following an increase in the particle concentration. Moreover, the link between the interfacial assembly of the particles and the stability of the foam was investigated. Following fulfillment of the Gibbs stability criterion, the foam volume was maintained somewhat constant. The drainage behavior of the foams prepared with fluorinated Janus particles was effectively hindered following irreversible adsorption at the air–water interface. Compared with traditional foam systems, the amphiphilic fluorinated nanoparticles foam system has a better plugging capacity and enhanced oil recovery capacity. It is noted that due to the unique anisotropic structure of the amphiphilic fluorinated nanoparticles, they have potential as colloid surfactants for enhanced oil recovery applications.

## Figures and Tables

**Table 1:** Composition of four foam systems.

Foam Systems	Composition
traditional surfactants	0.4 wt % CAB
homogeneous modified particles	0.4 wt % H30
mixtures of particles and surfactants	0.3 wt % DDAB + 0.1 wt % T30
amphiphilic fluorinated nanoparticles	0.4 wt % amphiphilic nanoparticles

**Table 2:** Oil recovery capacity of the foam systems.

Injection Mode	Sand Pack	K (μm^2^)	Oil Saturation	Oil Recovery before Injection of Foam		Oil Recovery after Injection of Foam		Enhanced Oil Recovery
				Respectively	Total	Respectively	Total	
surfactants	high	0.615	72.6%	48.3%	34.7%	4.9%	45.8%	11.1%
	low	0.044	73.8%	21.2%		17.3%		
homogeneous particles	high	0.624	71.9%	47.6%	35.3%	11.0%	56.5%	21.2%
	low	0.046	73.1%	22.9%		31.4%		
mixtures of particles and surfactants	high	0.591	71.9%	47.8%	34.5%	9.9%	49.8%	15.4%
	low	0.044	72.5%	21.1%		20.9%		
amphiphilic particles	high	0.607	71.4%	48.6%	34.9%	16.3%	67.8%	32.9%
	low	0.045	73.4%	21.2%		49.4%		
